# Retraction Note: MEK1/2 regulate normal BCR and ABL1 tumor-suppressor functions to dictate ATO response in TKI-resistant Ph+ leukemia

**DOI:** 10.1038/s41375-026-03026-w

**Published:** 2026-06-24

**Authors:** Laura Mazzera, Manuela Abeltino, Guerino Lombardi, Anna Maria Cantoni, Stefano Jottini, Attilio Corradi, Micaela Ricca, Elena Rossetti, Federico Armando, Angelo Peli, Anna Ferrari, Giovanni Martinelli, Maria Teresa Scupoli, Carlo Visco, Massimiliano Bonifacio, Alessia Ripamonti, Carlo Gambacorti-Passerini, Antonio Bonati, Roberto Perris, Paolo Lunghi

**Affiliations:** 1 Istituto Zooprofilattico Sperimentale della Lombardia e dell’Emilia Romagna “Bruno Ubertini”, Brescia, Italy; 2 Department of Medicine and Surgery, University of Parma, Parma, Italy; 3 Department of Veterinary Science, University of Parma, Parma, Italy; 4 National Healthcare Service (SSN—Servizio Sanitario Nazionale) ASL Piacenza, Piacenza, Italy; 5 University of Veterinary Medicine Hannover, Foundation, Hanover, Germany; 6 Department for Life Quality Studies Alma Mater Studiorum—University of Bologna, Bologna, Italy; 7 IRCCS Istituto Romagnolo per lo Studio dei Tumori (IRST) “Dino Amadori”, Meldola, FC Italy; 8 Institute of Hematology “L. e A. Seragnoli”, Department of Experimental, Diagnostic and Specialty Medicine, University of Bologna, Bologna, Italy; 9 Department of Neurosciences, Biomedicine and Movement Sciences, University of Verona, Verona, Italy; 10 Department of Engineering for Innovation Medicine, Section of Hematology—University of Verona, Verona, Italy; 11 Department of Medicine and Surgery, University of Milan-Bicocca, Monza, Italy; 12 Adult Hematology, IRCCS San Gerardo, Monza, Italy; 13 Department of Chemistry, Life Sciences and Environmental Sustainability, University of Parma, Parma, Italy; 14 Centre for Molecular and Translational Oncology—COMT, University of Parma, Parma, Italy

Retraction Note to: *Leukemia* 10.1038/s41375-023-01940-x, published online 29 June 2023

The authors have retracted this article. After publication, concerns were raised regarding similarities in some of the blots presented in Figs. 2 and 5. The authors have checked the data and found that a number of blots were incorrectly selected for the figures.

The authors intend to submit a revised version of the manuscript for peer review in due course.

Author Anna Maria Cantoni is deceased. All other authors agree with this retraction.

